# Diversity of the diet is correlated with osteoporosis in post-menopausal women: an Iranian case-control study

**DOI:** 10.3389/fpubh.2024.1431181

**Published:** 2024-08-09

**Authors:** Behnood Abbasi, Mohammad Mahdi Hajinasab, Zahra Mohammadi Zadeh, Paniz Ahmadi

**Affiliations:** ^1^Department of Nutrition, Electronic Health and Statistics Surveillance Research Center, Science and Research Branch, Islamic Azad University, Tehran, Iran; ^2^Department of Nutrition, Science and Research Branch, Islamic Azad University, Tehran, Iran

**Keywords:** osteoporosis, postmenopausal, postmenopausal osteoporosis, diet diversity score, bone resorption

## Abstract

**Background:**

Proper nutrition is a crucial factor in preventing osteoporosis, a significant pathological cause linked to skeletal weakness; this study investigated the relationship between dietary diversity score and food group diversity score with osteoporosis in postmenopausal women.

**Methods:**

This case-control study was conducted on 378 menopausal women aged 45–85 in Tehran, Iran. The age-matching method to control the confounding effect of age was used. The method of dual-energy X-ray absorptiometry (DXA) was used for assessing the bone mineral density of lumbar vertebrae and femoral neck. The bone mass status was evaluated with WHO criteria. All subjects were divided into the osteoporosis group and the non-osteoporosis group according to their T-score. A convenience sampling method was utilized to select the participants, which included two groups: case (*n* = 189) and control (*n* = 189). Data was collected using demographic and anthropometric information questionnaires, a valid 147 item food frequency questionnaire, and a physical activity questionnaire. Statistical analyses were conducted using SPSS-26, and *p*-values less than 0.05 were deemed to be statistically significant.

**Results:**

The results indicated significant differences in weight, body mass index, physical activity, smoking, and alcohol use between the two groups. The mean ± standard deviation of dietary diversity score (DDS) was lower in participants with osteoporosis (case) (3.31 ± 1.26) than in control (4.64 ± 1.33) (*p* < 0.001). The mean ± standard deviation of diversity score of cereals, fruits, and vegetables in the osteoporosis group (respectively: 0.71 ± 0.21, 0.94 ± 0.76, and 0.45 ± 0.44) was less than the control group (respectively: 0.80 ± 0.21, 1.64 ± 0.55 and 0.87 ± 0.42) (*p* < 0.001). After adjusting the confounding variables, the risk of osteoporosis had an inverse relationship with the diversity score of vegetable (OR = 0.16; 95%CI: 0.07–0.35), bread and cereal (OR = 0.21; 95% CI: 0.05–0.87) and fruit (OR = 0.35; 95%CI: 0.22–0.56) (*p* < 0.05). Nevertheless, no discernible correlation was seen between the tertiles of DDS, dairy and meat diversity score, and osteoporosis.

**Conclusion:**

We found a correlation between the diversity score of fruits, vegetables, and grains and osteoporosis. However, there is no significant correlation between the DDS triads and the diversity score of dairy products and meats with osteoporosis.

## Graphical abstract

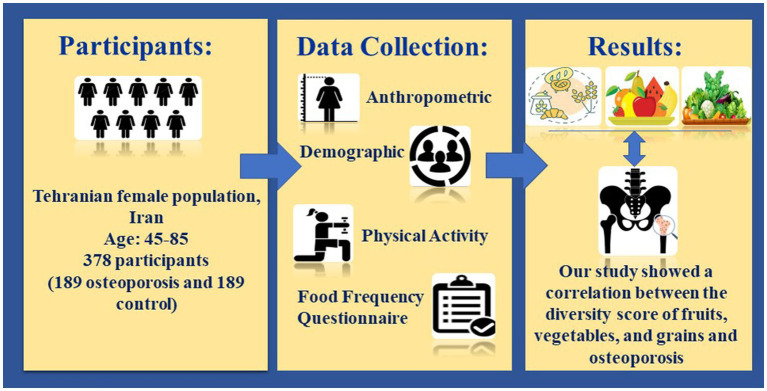

## Introduction

Systemic skeletal diseases such as osteoporosis raise the risk of bone fragility and fracture, increase the expense of medical treatment, and reduce bone mass and microarchitecture (1). It is one of the most common bone diseases in women over 55 and men over 65 (2). Osteoporosis fractures are estimated to affect 50% of women and 20% of men over the age of 50 (3, 4). The prevalence of osteoporosis has been steadily increased since 1999 (5–7). In Iran, a meta-analysis study in 2022 indicated that osteoporosis prevalence in postmenopausal women was 33.70% (8). In general, the risk factors for osteoporosis include age older than 65 years, history of systemic glucocorticoid use for more than 3 months, primary hyperparathyroidism, hypogonadism, and menopause before 45 years old, insufficient vitamin D and calcium intake, smoking, and a weight of less than 57 kg (9–13). Nutrition is an influential factor in osteoporosis, effectively creating peak bone mass (PBM) in childhood and adolescence and reducing bone loss in later years (14). In addition to the role of minerals, vitamins, proteins, and fats, the ratio of these substances is also effective in bone health (14, 15). Dietary diversity score (DDS) and food group diversity score (FGDS) are indicators which show the adequacy of nutrients (16) and quality of diet (17, 18). Generally, dietary diversity is measured through a questionnaire at the household or individual level; dietary diversity score (DDS) at the household level indicates the adequacy of food intake; at the individual level, the questionnaire provides information about the quality of the diet and nutrient intake (19, 20). Studies have shown that the higher the DDS, the lower the risk of some age-related diseases, including cognitive impairment, memory, diabetes and high blood pressure (21–25). Previous studies investigated the relationship between the dietary antioxidant index (DAI) and lacto-vegetarian dietary score (LVDS) with osteoporosis (26, 27). Also as mentioned, nutrition is one of the factors affecting osteoporosis (28), and postmenopausal osteoporosis is increasing among older adult women as the world’s demographics change (29). Moreover, regarding the association between DDS and age-related diseases and the limited number of studies on the effect of dietary diversity on osteoporosis, the aim of this research was to investigate the relationship between DDS and FGDS with osteoporosis in postmenopausal women.

## Methods

### Study population

This case-control study was performed in Tehran, Iran. Sample size using Gpower 3.1.9.2 software (30) and *F* test with linear multiple regression formula, with *R*^2^ deviation from zero (*α* = 0.05, power = 0.95, effect size = 0.1, *β* = 0.05), 176 subjects were calculated. Considering a dropout rate of 10% of the participants, the information of at least 189 people in each group was collected. A convenience sampling method was utilized to select the participants. In this research, we used the age-matching method to control the confounding effect of age. The method of dual-energy X-ray absorptiometry (DXA) was used for assessing the BMD of lumbar vertebrae and femoral neck. The bone mass status was evaluated with WHO criteria (T-score more than −1: normal BMD, T-score between −1 and −2.5: osteopenia, and T-score equal to or less than −2.5: osteoporosis) (31, 32). The diagnosis of osteoporosis case group, was confirmed by a rheumatology specialist.

**Figure 1:**
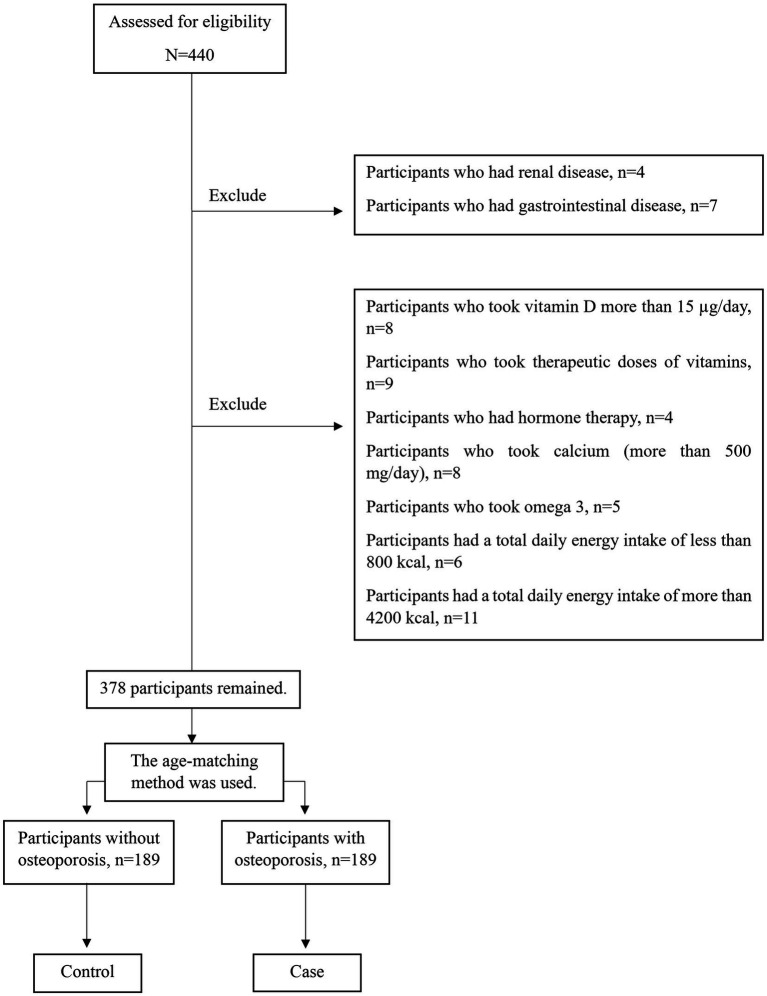
Graph of methodology.

All subjects were divided into the osteoporosis group and the non-osteoporosis group according to their T-score. In general, 378 postmenopausal women (189 cases and 189 controls) aged 45–85 who met the eligibility criteria were selected randomly from those referred to Shariati Hospital, private clinics, and health centers. Also, the control group was randomly selected from the women who were with the patients and met the study’s entry criteria (Figure 1). All participants were provided with a clear explanation of the research objectives and afterward signed written consent. Then, the participants’ information was gathered by a qualified expert. Menopause was described as a lack of the menstrual period throughout at least 12 months. The Biomedical Research Ethics Committee of Islamic Azad University-Science and Research Branch in Tehran, Iran, approved the research. (IR.IAU.SRB.REC.1396.119).

### Inclusion and exclusion criteria

The inclusion criteria included.

Not following a specific diet during the past year; not taking supplements or drugs that influence the bone metabolisms such as anticoagulants (33), glucocorticoids (34, 35), thyroxin (36), calcitonin (37, 38), antacids (39), Vitamin D (more than 15 μg/day) and calcium (more than 500 mg/day) (40, 41), consumption of therapeutic doses of vitamins or minerals (42), glucosamine (43, 44), omega-3 (45), and bisphosphonate (46); not have been diagnosed with endocrine, rheumatoid, hormone therapy, gastrointestinal, or renal diseases which effect density of bone mineral status.

The exclusion criteria were as follows.

Individuals who did not answer more than 20% of the questions of the Food Frequency Questionnaire (FFQ) and women with a Total daily energy intake of less than 800 kcal and more than 4,200 kcal (27, 47–50).

### Data collection

All the participants completed the valid questionnaires through the interviews, and an expert nutritionist evaluated all measurements. The general questionnaire gathered data about age, education, alcohol drinking, breastfeeding, and taking contraceptives. Additionally, a valid physical activity questionnaire was performed to estimate the physical activity status that was prepared in Europe, and its validity was approved by Daily Activity Questionnaire (51). The results were expressed in metabolic equivalent hours per day (Met-h/day) (51). The validity and reliability of this questionnaire were confirmed in Iran (52).

Body weight was calculated using digital scales (Tefal) after the participants wore lightweight clothing. Body weight was recorded within 100 g (0.1 kg) of precision. The height was assessed by a tape meter and was reported within 0.1 cm of accuracy while the contributors were standing and removing their shoes. Body mass index (BMI) was calculated as weight divided by height squared (kg/m^2^).

### Assessment of dietary intake

The participants’ dietary intake was obtained by a 147-item FFQ (53), which has been validated for validity and reliability in Iran (53). It was shown that the FFQ used has reasonable relative validity and reliability for nutrient intakes in Iranian adults and be an acceptable tool for assessing nutrient intakes in Iranian population (53). It assesses the frequency of consumption of each food item for the previous year. The Nutritionist IV program, specifically tailored for Iranian cuisine, was employed to convert the frequency of each food item in the FFQ to its corresponding weight in grams per day.

### Assessment of the dietary diversity score

DDS was calculated using the methodology proposed by Kant et al. (54). According to the Food Guide Pyramid published by the USDA (55), foods were divided into five groups: bread and cereals, meat and eggs, dairy, vegetables, and fruits. The five groups were categorized into 23 subgroups, comprising seven subgroups in the bread and cereals group (refined bread, biscuits, pasta, whole-bread, breakfast cereals, rice, and refined flour), four subgroups in the meat and eggs group (red meat, chicken, fish, and eggs), three subgroups in the dairy group (milk, yoghurt, and cheese), seven subgroups in the vegetable group (vegetables, potatoes, tomatoes, other starchy vegetables, legumes, yellow vegetables), and two subgroups in the fruit group (fruits and juices).

Each group has a maximum 2-point diversity score (FGDS ≤ 2). The final DDS is equal to the summation of five FGDSs. Consequently, the maximum DDS is 10. The DDS tertiles were also categorized for further analysis with cutoff points of 3.2 and 4.5. The FGDS was computed by dividing the total of consumed subgroups by the number of subgroups and then multiplying the result by two. A subset was only considered a consumer if the participant consumed at least half of the servings per day of that subgroup. For example, if a participant has consumed two subgroups of vegetables, the FGDS of this group will equal the numerator 2/7×2=0.57 . So, this participant gives only a 0.57 score for the vegetable group (vegetable FGDS = 0.57).

### Statistical analysis

Data analysis was conducted using SPSS software version 26. A *p*-value of ≤0.05 was considered as statistically significance. This research characterized the qualitative variables in terms of their frequency expressed as a percentage. The Chi-square test was used to assess qualitative variables. The mean, together with its corresponding standard deviation, was used to characterize the quantitative variables. The Kolmogorov–Smirnov test was used to examine the normal distribution of the data. To examine the quantitative variables between the osteoporosis and control groups, the independent t-test was used for variables that followed a normal distribution, while the Mann–Whitney-U test was utilized for variables that did not adhere to a normal distribution. Multivariable logistic regression was used to assess the relationship between DDS and, FGDS, and osteoporosis. In multivariable-adjusted models, data were controlled for the confounders, including demographic and anthropometric characteristics of participants.

## Results

### Demographic, anthropometric characteristics and physical activity

Demographic and anthropometric characteristics between osteoporosis and control groups are shown in Table 1. There were significant differences in weight (*p* = 0.001), BMI (*p* < 0.001), physical activity (*p* < 0.001), smoking (*p* < 0.001), and alcohol (*p* < 0.001) consumption between the osteoporosis and control groups. There were no significant differences in age (*p* = 0.781), marital status (*p* = 0.833), history of twinning (*p* = 0.174), breast-feeding (*p* = 0.148), using birth control pills (*p* = 0.345), lactation period (*p* = 0.135), number of pregnancies (*p* = 0.246), age of first pregnancy (*p* = 0.115), duration of contraceptive use (*p* = 0.305), last time of contraceptive use (*p* = 0.202), and education between the two groups.

**Table 1:** Demographic and anthropometric characteristics of the two study groups of participants.

Variables		Case	Control	*p*-value
Quantitative variables				
Age (years)		54.53 ± 5.35	54.6 ± 5.11	0.781^b^
Weight (kg)		75.4 ± 12.3	71.5 ± 9.9	**0.001** ^**a**^
Height (cm)		160.60 ± 5.52	160.17 ± 7.65	0.70^b^
BMI (kg/m^2^)		29.22 ± 4.29	28.05 ± 5.42	**<0.001** ^**a**^
Number of pregnancies		3.00 ± 1.36	3.11 ± 1.26	0.246^b^
Age of first pregnancy		21.48 ± 4.89	20.66 ± 5.16	0.115^b^
Lactation period		30.51 ± 25.85	35.07 ± 32.41	0.135^b^
Duration of taking OCP		16.71 ± 32.77	13.31 ± 27.47	0.305^b^
Last time to take OCP		3.95 ± 6.76	3.84 ± 7.47	0.202^b^
PA (MET-hour-week)		1,549.65 ± 821.1	2,298.85 ± 2,158.1	**<0.001** ^**b**^
Categorical variable				
Marital status	Married	178 (94.2%)^b^	176 (93.1%)	0.833^c^
	Single	11 (5.8%)	13 (6.9%)	
History of twining	Yes	7 (3.7%)	2 (1.1%)	0.174^c^
	No	182 (96.3%)	187 (98.9%)	
Breast-feeding	Yes	172 (91%)	162 (85.7%)	0.148^c^
	No	17 (9%)	27 (14.3%)	
Smoking	Yes	32 (16.9%)	0 (0.00%)	**<0.001** ^**c**^
	No	157 (83.1%)	189 (100%)	
Alcohol	Yes	25 (13.2%)	4 (2.1%)	**<0.001** ^**c**^
	No	164 (86.8%)	185 (97.9%)	
OCP	Yes	67 (35.4%)	57 (30.2%)	0.345^c^
	No	122 (64.6%)	131 (69.3%)	
Education	Undergraduate	156 (82.5%)	138 (73%)	0.071^c^
	Graduate	32 (16.9%)	48 (25.4%)	
	Postgraduate	1 (0.5%)	3 (1.6%)	

OCP, Oral Contraceptive Pills; BMI, Body mass index; PA, physical activity; METs, Metabolic Equivalents.

^a^Resulted from independent t-test for quantitative variables; ^b^Resulted from Mann–Whitney U test for quantitative variables; ^c^Resulted from chi-square test for categorical variables; *p*-values <0.05 was considered significant; Quantitative variables: mean ± SD; Qualitative variables: frequency (percentage). *p*-values marked in bold show significant differences.

### DDS and FGDS of the two study groups of participants

Table 2 shows the DDS and FGDS of both the osteoporosis and control groups. The mean DDS and FGDS of bread and cereals, vegetables, and fruits significantly differed between the two groups (*p* < 0.001). Nevertheless, there was no substantial disparity in the average FGDS of meat (*p* = 0.89) and dairy products (*p* = 0.09) among the individuals in both groups.

**Table 2:** DDS and FGDS of the two study groups of participants.

Diversity score	Case^c^	Control^c^	*p*-value
Bread and cereals	0.71 ± 0.21	0.80 ± 0.21	**<0.001** ^**b**^
Meat	0.31 ± 0.35	0.33 ± 0.44	0.89^b^
Dairy	0.88 ± 0.52	0.98 ± 0.51	0.09^b^
Vegetables	0.45 ± 0.44	0.87 ± 0.42	**<0.001** ^**b**^
Fruit	0.94 ± 0.76	1.64 ± 0.55	**<0.001** ^**b**^
DDS	3.31 ± 1.26	4.64 ± 1.33	**<0.001** ^**a**^

DDS, Dietary Diversity Score.

^a^Resulted from independent t-test; ^b^Resulted from Mann–Whitney U test; ^c^Quantitative variables: mean ± SD; *p*-value <0.05 was considered significant. *p*-values marked in bold show significant differences.

### Dietary intake of the two study groups of participants

Table 3 illustrates the dietary intake of participants in both study groups. The control group consumed significantly more total protein (*p* = 0.003), low-fat dairy products (*p* < 0.001), and eggs (*p* < 0.001) than the osteoporosis group. The osteoporosis group consumed significantly more total fat (*p* < 0.001), saturated fat (*p* < 0.001), Fatty dairy products (*p* = 0.001), refined carbohydrates (*p* < 0.001), and processed meats (*p* = 0.003) compared to the control group. Total carbohydrate and energy consumption were not notably different between the two groups (*p* > 0.05).

**Table 3:** Dietary intake of the two study groups of participants.

Dietary intakes	Case^c^	Control^c^	*p*-value
Energy intake (kcal)	2,791.7 ± 884.4	2,661.3 ± 893.1	0.814^b^
Total protein (g)	85 ± 25.4	92.9 ± 33.4	**0.003** ^**a**^
Total carbohydrates (g)	360.5 ± 111.4	374.7 ± 123.3	0.432^b^
Refined carbohydrates (g)	347.4 ± 121.9	238.8 ± 122.3	**<0.001** ^**b**^
Total fat (g)	110 ± 44.7	81.2 ± 31.7	**<0.001** ^**b**^
Dairy products (g)	219.3 ± 218.1	159.5 ± 185.2	**0.001** ^**b**^
Low-fat dairy products (g)	116.4 ± 139.3	302.7 ± 261	**<0.001** ^**b**^
Saturated fats (g)	31.1 ± 29.5	11.8 ± 18.4	**<0.001** ^**b**^
Processed meat (g)	14 ± 26.4	5.4 ± 33.1	**0.003** ^**b**^
Egg (g)	11.7 ± 17.9	26.3 ± 26.6	**<0.001** ^**b**^

^a^Resulted from independent t-test; ^b^Resulted from Mann–Whitney U test; ^c^Quantitative variables: mean ± SD; *p*-value <0.05 was considered significant. *p*-values marked in bold show significant differences.

### Odds ratio of osteoporosis

Table 4 shows the odds ratios (OR) and 95% confidence intervals (CI) for the association between the dietary diversity score (DDS) and the food group diversity score (FGDS) with osteoporosis after adjusting for multiple variables. In the crude model, we found that the risk of getting osteoporosis decreased by increasing the FGDS of vegetables (OR = 0.262; 95% CI: 0.14–0.48) and fruits (OR = 0.329; 95% CI: 0.22–0.48). After adjusting the effect of confounding variables (Model 3), it was also found that increasing the FGDS of bread and cereals (OR = 0.219; 95% CI: 0.05–0.87) was likely to have lower the risk of having osteoporosis. Compared to the crude model, this risk was reduced in vegetables (OR = 0.165; 95% CI: 0.07–0.35), but there was no such a result in fruits (OR = 0.354; 95% CI: 0.22–0.56). However, no significant relationship was observed between DDS tertiles 1 and 2 compared to 3, meat and dairy diversity scores with osteoporosis (*p* > 0.05).

**Table 4:** Crude and multivariable-adjusted OR and 95% CI for the association of DDS and FGDS and osteoporosis.

		Crud model		Model^1^		Model^2^		Model^3^	
		OR (CI)^a^	*p*-value	OR (CI)^a^	*p*-value	OR (CI)^a^	*p*-value	OR (CI)^a^	*p*-value
Bread and cereals		0.427 (0.14–1.30)	0.135	0.313 (0.09–1.00)	0.052	0.312 (0.08–1.11)	0.073	0.219 (0.05–0.87)	**0.031**
Meat and eggs		1.278 (0.67–2.42)	0.453	1.122 (0.56–2.23)	0.743	1.074 (0.49–2.31)	0.854	0.873 (0.38–1.98)	0.746
Dairy		0.860 (0.53–1.37)	0.529	0.783 (0.47–1.28)	0.336	0.884 (0.51–1.52)	0.656	0.948 (0.52–1.70)	0.858
Vegetable		0.262 (0.14–0.48)	**<0.001**	0.272 (0.14–0.52)	<0.001	0.210 (0.10–0.43)	<0.001	0.165 (0.07–0.35)	**<0.001**
Fruit		0.329 (0.22–0.48)	**<0.001**	0.381 (0.25–0.56)	<0.001	0.381 (0.25–0.58)	<0.001	0.354 (0.22–0.56)	**<0.001**
DDS Tertile	1 vs. 3	1.003 (0.56–1.78)	0.992	0.773 (0.39–1.52)	0.458	1.041 (0.49–2.17)	0.916	0.998 (0.44–2.24)	0.996
	2 vs. 3	0.824 (0.46–1.47)	0.514	0.740 (0.39–1.39)	0.353	0.968 (0.48–1.94)	0.926	1.122 (0.51–2.46)	0.774

^a^Based on logistic regression, values were shown presented as Odds Ratios (OR) with 95% Confidence Intervals (CI).

^1^Adjusted for weight, BMI, and physical activity.

^2^Adjusted for weight, BMI, physical activity, smoking, and alcohol.

^3^Adjusted for weight, height, BMI, marital status, history of twins, number of pregnancies, age of first pregnancy, Breastfeeding, Breastfeeding period, smoking, alcohol, oral contraceptive pills, duration of taking oral contraceptive pills, Last time to take oral contraceptive pills, level of education, and physical activity.

*p*-value <0.05 was considered significant. *p*-values marked in bold show significant differences.

## Discussion

The present study investigates the relationship of the DDS and FGDS to osteoporosis in Iranian postmenopausal women. Previous studies show that DDS is a suitable indicator for the adequacy of dietary intake and diet quality (16, 56). Considering the effect of nutrients and diet quality on the risk of developing osteoporosis, it seems that DDS and the scores of various food groups correlate with osteoporosis (57). DDS is associated with better health status (58). Research has indicated that an increased DDS is associated with a decreased likelihood of developing age-related ailments such as cognitive and memory impairments (22, 59), diabetes (60), and hypertension (61). Also, Liyuan Tao and Minatsu Kobayashi et al. demonstrated that individuals with a higher DDS have a reduced probability of mortality (62, 63).

We found that the risk of osteoporosis in postmenopausal women would reduce as the diversity score of fruits and vegetables rose. Consumption of minerals such as calcium, antioxidant vitamins such as vitamins C and E, flavonoids, and carotenoids are known as dietary factors that prevent the reduction of bone mineral density and osteoporosis (64, 65). Vegetables and fruits contain considerable amounts of nutrient-dense that are very important for bone health. Antioxidants such as polyphenols and phytoestrogens are helpful for bone health because of their anti-inflammatory properties (66, 67). Vitamin C, found in fruits and vegetables, is an antioxidant and cofactor for collagen synthesis, enhancing bone formation and protecting against oxidative damage, which can prevent osteoporosis (68–70). Besides, vitamin C can help maintain osteoblast differentiation markers (such as Osterix, osteocalcin, runt-related transcription factor 2, and bone morphogenetic protein 2), reduce bone loss, and promote bone formation (71).

Vitamin K, found in vegetables (such as dark green leafy vegetables), activates osteocalcin (a protein that binds calcium to the bone matrix), increasing bone mineral density (72). Furthermore, Vitamin K is crucial for maintaining bone strength by activating bone proteins like matrix Gla protein (MGP), Gla-rich protein, protein S, and growth arrest-specific 6 protein (Gas6) (73). Mangano et al. showed that high consumption of fruits and vegetables is related to bone health and reduces fractures in adults (74). Fruits and vegetables change the metabolic pathways of bones by affecting the intestinal microbiome (74–76). Also, fruits and vegetables positively affect women’s bone health by reducing the level of inflammatory compounds, absorbing osteoclasts, differentiating osteoblasts, and increasing estrogen (74, 77, 78). Fruits and vegetables, as sources of alkaline precursors (for example, K, Ca, Mg), can neutralize the effects of an acidic diet on bone tissue and, as a result, reduce bone resorption and increase bone density (79).

In our study, after adjusting the effect of confounding variables, a significant relationship was observed between the variety score of bread and cereals with osteoporosis. Studies have shown the positive effect of diets containing whole grains and the negative effect of simple sugars on reducing the risk of osteoporosis (80, 81). Muñoz-Garach and Ilesanmi-Oyelere et al. have shown that simple sugars increase the risk of osteoporosis by increasing inflammation, hyperinsulinemia, increasing renal acid load, decreasing calcium intake, and increasing calcium excretion (29, 82). However, whole grains positively affect bone health due to the presence of magnesium, iron, phytochemicals, and antioxidants (83).

Within our research, there was no significant relationship between the diversity score of the dairy group and osteoporosis. In fact, dairy products are a rich source of protein, calcium, phosphorus, and potassium; along with vitamin D, calcium affects bone formation and metabolism (84, 85). Phosphorus and potassium help bone mineralization by strengthening the natural metabolism of calcium (86, 87). Milk proteins increase the activity of osteoblasts, stimulate bone mineralization, and, as a result, improve the condition of bones (88). However, the results regarding the effect of dairy products on osteoporosis are contradictory. Yingjie Shi et al., in a meta-analysis study conducted in 2020, showed that dairy products improve bone mineral density in healthy postmenopausal women and help prevent osteoporosis (89); while, in another meta-analysis study, Esmaillzadeh et al. showed that milk consumption and dairy products are not associated with a reduced risk of osteoporosis and bone fractures (90). Karl Michaëlsson et al. showed that milk is the main dietary source of D-galactose; d-galactose exposure in animals, with a dose corresponding to the recommended amount of milk in humans, induces oxidative stress damage and chronic inflammation; in addition, excess of galactose reacts non-enzymatically with amino groups in proteins and peptides forming advanced glycation end products (AGEs) (91). AGEs could disrupt the functions of osteoblasts by inducing cell ferroptosis (92). Also, considering that studies have shown that dairy products do not have the same effect on bones (93–95). It is possible that considering both types of low-fat and Fatty dairy products as subgroups of the diversity of dairy products is the cause of this result. For this purpose, we compared the average consumption of low-fat and high-fat dairy products between two groups with and without osteoporosis. The results showed that there is a significant difference between the mean of low-fat and high-fat dairy products consumed by two groups, so that in people with osteoporosis, most of the consumed dairy products were in high-fat form and in healthy people in low-fat form.

We observed no significant association between the meat diversity scores with osteoporosis. Although some studies show a positive relationship between protein intake and bone health (66), but some studies suggest that high protein consumption can lead to increased bone loss, possibly due to the acidic nature of a high-protein diet (66). Other studies show the positive effect of eggs and the negative effect of processed meats on bone health (96, 97). Kari Martyniak et al. have shown that the saturated fatty acids in red meat, viscera, and processed meats prevent bone repair (98). The comparison of subgroups of meat diversity scores shows that the average consumption of eggs in healthy people is higher than that of people with osteoporosis; in other words, the consumption of processed meats is higher in people with osteoporosis than in healthy people. As a result, considering the same score for processed meats and eggs to calculate the meat variety score and evaluate the relationship between meat variety and osteoporosis is not appropriate.

In our study, there was no significant relationship between DDS triads and osteoporosis. In the study by Jian Zhang, there was an inverse relationship between DDS and bone fracture in people 60 years old and younger (99). While in a study by Kwon and Lee, in the same direction as our study, the lowest quintile compared to the highest quintile of DDS was not associated with the risk of osteoporosis in the age group of 50–64 years (64). Some studies have shown that the increase in DDS, in addition to being related to the consumption of healthy foods and the intake of nutrients, is also related to the intake of unhealthy foods (100), on the other hand, studies show the positive effect of fruits, vegetables, nuts and legumes, low-fat dairy products, and whole grains, and the negative effect of sodium consumption, sweets, and red or processed meat, the reason for these differences can be considered due to the effect of unhealthy foods in the calculation of DDS.

The osteoporosis is a major public health problem (101). We recommended the implementation of osteoporosis prevention screening programs for early diagnosis. This would be coupled with educational initiatives promoting healthy dietary and systematic resistance training as strategies to mitigate osteoporosis risk in postmenopausal women. Furthermore, we recommend that prospective studies investigate the effects of DDS and FGDS across diverse age groups and cultural backgrounds. Their effect on bone circulation markers should also be considered. Additionally, it is recommended to evaluate diversity scores to avoid foods harmful to bone health (such as processed meats and Fatty dairy products).

### Strengths and limitations

As far as we know, it is the first case-control study in the country to investigate the relationship between DDS and diversity score of food groups with osteoporosis. To minimize information bias, we used validated Food Frequency Questionnaires (FFQ), which could correctly describe previous long-term dietary intake, and Metabolic Equivalent of Task (MET) questionnaires for data collection. We evaluated a sufficient sample size and adjusted for confounders based on previous studies. We used the age-matching method to control the confounding effect of age.

Nevertheless, this study had some limitations. Due to the difference in dose supplementation and absorption and the results of previous studies, the considered non-therapeutic doses of vitamin or mineral amounts have not been questioned. Furthermore, selecting a greater number of samples can potentially enhance the validity of the research outcomes. Also, we did not have enough information on sun exposure time for individuals. Samples were collected from a variety of urban areas, and the quality of life in these areas can be influenced by many factors. It is essential to consider the possibility of error in people’s reporting, as the FFQ method relies on memory. Besides, due to the variations in food culture, the availability of food, and the different cooking methods across different countries, comparing their DDS could lead to potential errors in the studies. Given the racial differences, the results of this study can be generalized to Middle Eastern countries. However, it is important to consider the study’s limitations when interpreting these findings.

## Conclusion

In summary, we found an association between fruit, vegetable, and grain diversity scores and osteoporosis. However, no detectable association was found between DDS triads and the osteoporosis-related dairy and meat variety score. Therefore, based on the observed likelihood correlation, it is recommended to increase the diversity score in the consumption of bread and cereals, fruits, and vegetables in the diet of menopausal women. Many more studies are needed to confirm this relationship, and especially to evaluate the causal relationship in this field, randomized clinical trial studies (RCTs) are necessary.

## Data availability statement

The raw data supporting the conclusions of this article will be made available by the authors, without undue reservation.

## Ethics statement

The Biomedical Research Ethics Committee of Islamic Azad University-Science and Research Branch in Tehran, Iran, approved the research (IR.IAU.SRB.REC.1396.119). All participants signed a written informed consent form approved by the Ethics Committee.

## Author contributions

BA: Methodology, Project administration, Supervision, Validation, Writing – original draft, Writing – review & editing, Data curation. MH: Writing – original draft, Writing – review & editing. ZM: Writing – original draft. PA: Writing – review & editing.
